# In search of the genetic footprints of Sumerians: a survey of Y-chromosome and mtDNA variation in the Marsh Arabs of Iraq

**DOI:** 10.1186/1471-2148-11-288

**Published:** 2011-10-04

**Authors:** Nadia Al-Zahery, Maria Pala, Vincenza Battaglia, Viola Grugni, Mohammed A Hamod, Baharak Hooshiar Kashani, Anna Olivieri, Antonio Torroni, Augusta S Santachiara-Benerecetti, Ornella Semino

**Affiliations:** 1 Dipartimento di Genetica e Microbiologia, Università di Pavia, Via Ferrata 1, 27100 Pavia, Italy; 2 Department of Biotechnology, Faculty of Sciences, Baghdad University, 10001 Baghdad, Iraq; 3 Department of Microbiology, UNESCO-MIRCEN for Marine Biotechnology, College of Fisheries, 575002 Mangalore, India; 4 Centro Interdipartimentale "Studi di Genere", Università di Pavia, 27100 Pavia, Italy

## Abstract

**Background:**

For millennia, the southern part of the Mesopotamia has been a wetland region generated by the Tigris and Euphrates rivers before flowing into the Gulf. This area has been occupied by human communities since ancient times and the present-day inhabitants, the Marsh Arabs, are considered the population with the strongest link to ancient Sumerians. Popular tradition, however, considers the Marsh Arabs as a foreign group, of unknown origin, which arrived in the marshlands when the rearing of water buffalo was introduced to the region.

**Results:**

To shed some light on the paternal and maternal origin of this population, Y chromosome and mitochondrial DNA (mtDNA) variation was surveyed in 143 Marsh Arabs and in a large sample of Iraqi controls. Analyses of the haplogroups and sub-haplogroups observed in the Marsh Arabs revealed a prevalent autochthonous Middle Eastern component for both male and female gene pools, with weak South-West Asian and African contributions, more evident in mtDNA. A higher male than female homogeneity is characteristic of the Marsh Arab gene pool, likely due to a strong male genetic drift determined by socio-cultural factors (patrilocality, polygamy, unequal male and female migration rates).

**Conclusions:**

Evidence of genetic stratification ascribable to the Sumerian development was provided by the Y-chromosome data where the J1-Page08 branch reveals a local expansion, almost contemporary with the Sumerian City State period that characterized Southern Mesopotamia. On the other hand, a more ancient background shared with Northern Mesopotamia is revealed by the less represented Y-chromosome lineage J1-M267*. Overall our results indicate that the introduction of water buffalo breeding and rice farming, most likely from the Indian sub-continent, only marginally affected the gene pool of autochthonous people of the region. Furthermore, a prevalent Middle Eastern ancestry of the modern population of the marshes of southern Iraq implies that if the Marsh Arabs are descendants of the ancient Sumerians, also the Sumerians were most likely autochthonous and not of Indian or South Asian ancestry.

## Background

The Near East is well known for its important role in human history, particularly as a theatre for great historical events that changed the face of the world during the Neolithic period. The temperate climate and fertile soil brought by the continuous flooding of the Tigris and Euphrates rivers, made the Mesopotamian region ideal for early revolutions in agriculture and farming. In particular, the southern part of Mesopotamia (the delta between the two rivers in the present day southern Iraq) has been historically known as the Garden of Eden (biblical name) or Sumer Land, the land of Abraham.

The Mesopotamian civilization originated around the 4^th ^millennium BC in the low course of the Tigris and Euphrates rivers. This alluvial territory, which emerged progressively by soil sedimentation, attracted different populations from the northern and eastern mountains but, whereas traces of their culture are present in the territory, as documented by the Ubaid-Eridu pottery, nothing is available for their identification. Only two groups of populations arrived later and in larger number leaved historical records: Sumerian and Semitic groups. The Sumerians, who spoke an isolated language not correlated to any linguistic family, are the most ancient group living in the region for which we have historical evidence. They occupied the delta between the two rivers in the southern part of the present Iraq, one of the oldest inhabited wetland environments. The Semitic groups were semi-nomadic people who spoke a Semitic language and lived in the northern area of the Syro-Arabian desert breeding small animals. From here, they reached Mesopotamia where they settled among the pre-existing populations. The Semitic people, more numerous in the north, and the Sumerians, more represented in the south, after having adsorbed the pre-existing populations, melted their cultures laying the basis of the western civilization [1].

### Mesopotamia Marshes

**Figure 1:**
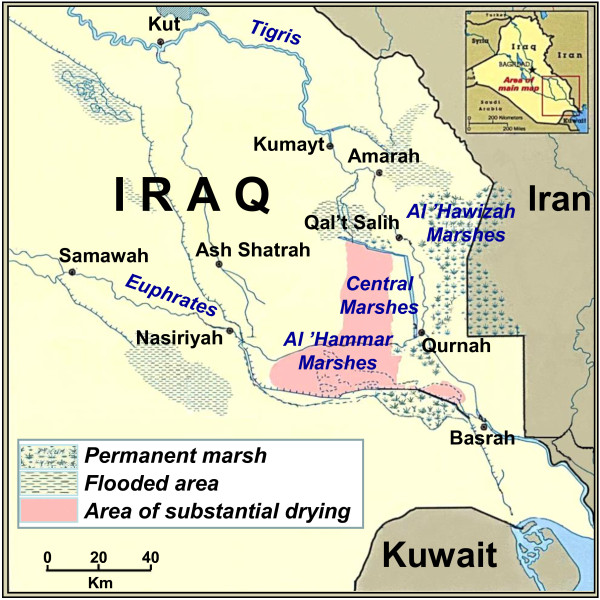
**Map of Iraq illustrating present and former Marsh areas**. The majority of the subjects analysed in this study are from the Al-Hawizah Marshes, the only natural remaining marsh area in southern Iraq [4].

The Mesopotamian marshes are among the oldest and, until twenty years ago, the largest wetland environments in Southwest Asia, including three main areas (Figure 1): the northern Al-Hawizah, the southern Al-Hammar and the so-called Central Marshes all rich in both natural resources and biodiversity [2,3]. However, during the last decades of the past century, a systematic plan of water diversion and draining drastically reduced the extension of the Iraqi marshes, and by the year 2000 only the northern portion of Al-Hawizah (about 10% of its original extension) remained as functioning marshland whereas the Central and Al-Hammar marshes were completely destroyed. This ecological catastrophe constrained Marsh Arabs of the drained zones to leave their niche: some of them moved to the dry land next to the marshes and others went in diaspora. However, due to the attachment to their lifestyle, Marsh Arabs have been returned to their land as soon as the restoration of marshes began (2003) [4].

The ancient inhabitants of the marsh areas were Sumerians, who were the first to develop an urban civilization some 5,000 years ago. Although footprints of their great civilization are still evident in prominent archaeological sites lying on the edges of the marshes, such as the ancient Sumerian cities of Lagash, Ur, Uruk, Eridu and Larsa, the origin of Sumerians is still a matter of debate [5]. With respect to this question, two main scenarios have been proposed: according to the first, the original Sumerians were a group of populations who had migrated from "the Southeast" (India region) and took the seashore route through Arabian Gulf before settling down in the southern marshes of Iraq [6]. The second hypothesis posits that the advancement of the Sumerian civilization was the result of human migrations from the mountainous area of Northeastern Mesopotamia to the southern marshes of Iraq [7], with ensuing assimilation of the previous populations.

Over time, the many historical and archaeological expeditions that have been conducted in the marshes have consistently reported numerous parallelisms between the modern and ancient life styles of the marsh people [8,9]. Details such as home architecture (particular arched reed buildings), food gathering (grazing water buffalos, trapping birds and spearing fish, rice cultivation), and means of transportation (slender bitumen-covered wooden boats, called "Tarada") are documented as still being practiced by the indigenous population locally named "Ma'dan" or "Marsh Arabs" [10,11]. This village life-style, which has remained unchanged for seven millennia, suggests a possible link between the present-day marsh inhabitants and ancient Sumerians. However, popular tradition considers the Marsh Arabs as a foreign group, of unknown origin, which arrived in the marshlands when the rearing of water buffalo was introduced to the region.

In order to shed some light on the origin of the ancient and modern Mesopotamian marsh populations, which remains ambiguous in spite of all the above mentioned theories, the genetic variation of a sample of "Marsh Arabs" has been investigated both for the maternally transmitted mitochondrial DNA (mtDNA) and the Male Specific region of the Y chromosome (MSY).

## Methods

### The sample

The sample consists of 143 healthy unrelated males, mainly from the Al-Hawizah Marshes (the only not drained South Iraqi marsh area[4] (Figure 1). For each subject the "Marsh Arab" ancestry (at least for last four generations) was ascertained by interview after having obtained the informed consent. The collection (5-10 ml of blood each, in EDTA) was carried out in different villages during a field expedition. DNA was extracted from whole blood by using a standard phenol/chloroform protocol. For comparison, a sample of 154 Iraqi subjects representative of the general Iraqi population and therefore referred throughout the text as "Iraqi" was investigated for both mtDNA and Y-chromosome markers. This sample, previously analysed at low resolution [12] is mainly composed of Arabs, living along the Tigris and Euphrates Rivers. In addition, the distribution of the Y-chromosome haplogroup (Hg) J1 sub-clades was also investigated in four samples from Kuwait (N = 53), Palestine (N = 15), Israeli Druze (N = 37) and Khuzestan (South West Iran, N = 47) as well as in more than 3,700 subjects from 39 populations, mainly from Europe and the Mediterranean area but also from Africa and Asia [13,14].

This research was approved by the Ethics Committee for Clinical Experimentation at the University of Pavia (Board minutes of October 5, 2010).

### Y-chromosome genotyping

**Figure 2:**
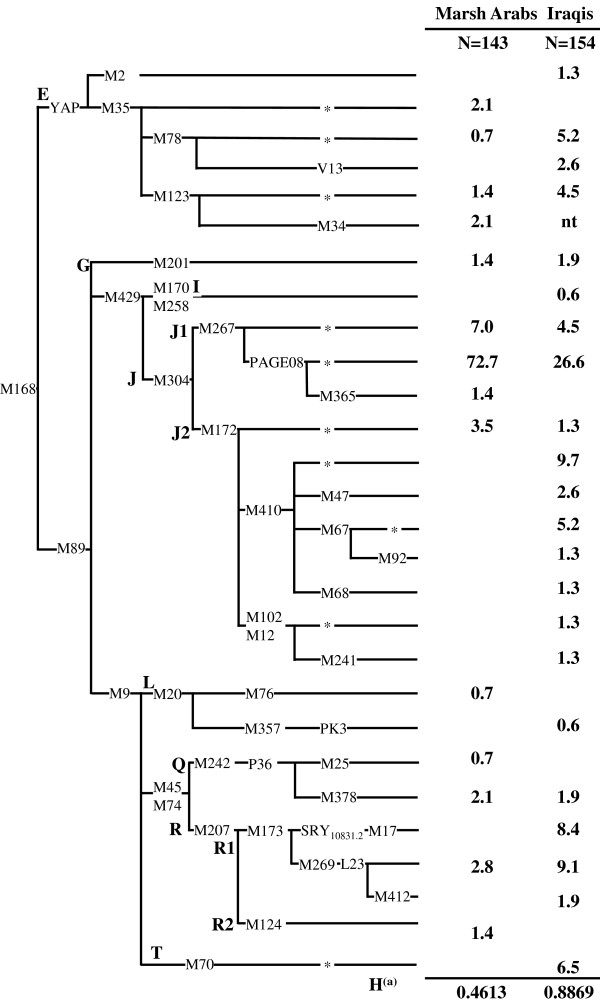
**Phylogeny of Y-chromosome haplogroups and their frequencies (%) in Marsh Arab and Iraqi populations**. Haplogroups are labelled according to the Y Chromosome Consortium [17,18] and the International Society of Genetic Genealogy [16]. Differently from previously reported [19], the M365 mutation was observed in two J1-Page08 Y-chromosomes (Marsh Arabs). In these two subjects, M365 was observed in association with the new mutation L267.2 discovered while typing the M365 marker. It consists of an A to G transition at nucleotide position 159. The markers P37, M253, M223 of haplogroup I, M81 and M293 of haplogroups E, and M367, M368 and M369 of haplogroup J1 were typed but not observed. A star (*) indicates a paragroup: a group of Y chromosomes not defined by any reported phylogenetic downstream mutation. nt: not tested. (a) Heterogeneity.

Y-chromosome haplogroup affiliation was determined according to the most recently updated phylogeny [15,16] by genotyping, in hierarchical order, 46 single nucleotide polymorphisms (SNPs) (see the phylogeny in Figure 2 of the Result section). The nomenclature was according to the Y chromosome Consortium rules [17-19].

Mutations were detected either as RFLPs or by DHPLC of pertinent fragments amplified by PCR [Additional file 1]. When necessary, results were verified by sequencing.

J1-M267 Y chromosomes were analysed for a panel of eight Y-chromosome microsatellite loci (DYS19, YCAIIa/b, DYS389I/II, DYS390, DYS391 and DYS392) by using multiplex reactions according to the STR DNA Internet Data Base [20] and fragment analysis by capillary electrophoresis on ABI PRISM^® ^3100 Genetic Analyzer.

### MtDNA genotyping

Affiliation within mtDNA haplogroups was first inferred through the sequencing of a fragment of 746-846 base pairs (bps) from the control region that, according to the rCRS [21], encompasses the entire hypervariable segment I (HVS-I) and part of HVS-II and then confirmed through a hierarchical survey by PCR-RFLP/sequencing of coding region haplogroup diagnostic markers. [Additional file 2]. The nomenclature was according to van Oven and Kayser, built 12 (20 July 2011) [22].

Sequence analysis of the control region was performed by using primers F15973 (5'- AACTCCACCATTAGCACCCA-3') and R263 (5'-TGGCTGTGCAGACATTCAAT-3') on amplicons obtained by using the primers F15877 (5'-CAAATGGGCCTGTCCTTGTA-3') and R468 (5'-GGAGTGGGAGGGGAAAATAA-3') [23]. All the samples were sequenced starting from primer F15973. Samples showing potential heteroplasmies or ambiguities were also sequenced with primer R263.

### Statistical analysis

Haplogroup diversity (H) was computed by using the standard method of Nei [24]. Principal component (PC) analysis was performed on haplogroup frequencies using Excel software implemented by XLstat. The relative amount of accumulated diversity, as a function of geography, was evaluated through the mean microsatellite variance estimated on at least five individuals. Haplogroup frequency and variance maps were generated by using Surfer software [25], following the Kriging procedure. Median-Joining (MJ) networks [26] were constructed by the Network 4.5.0.0 program [27]. MtDNA and Y-chromosome networks were obtained by the MJ method, with ε = 0 and weighting markers according to their relative stability (Y-chromosome: microsatellite loci were weighted proportionally to the inverse of the repeat variance of each haplogroup. MtDNA: coding region was weighted as four; control region as one - see Additional file 2) and after having processed the data with the reduced-median method. The age of microsatellite variation within haplogroups was evaluated according to Zhivotovsky et al. [28] and Sengupta et al. [29] and through the BATWING procedure (Bayesian Analysis of Trees With Internal Node Generation) [30]. Batwing runs used a model assuming an initial constant population size followed by expansion at time β with a growth rate of α. Alfa and beta parameters were as in Tofanelli et al. [31]. For both methods the effective mutation rate and a generation time of 25 years [28] were used.

## Results

### Y-chromosome variation

The screening of 45 SNPs, plus one identified in this survey, in Marsh Arabs and Iraqis identified 28 haplogroups, 14 in the marsh sample and 22 in the control Iraqis. Only eight haplogroups were shared by both groups. Their phylogenetic relationships and frequencies are shown in Figure 2. More than 90% of both Y-chromosome gene pools can be traced back to Western Eurasian components: the Middle Eastern Hg J-M304, the Near Eastern Hgs G-M201, E-M78 and E-M123, while the Eurasian Hgs I-M170 and R-M207 are scarce and less common in the Marsh Arabs than in the control sample. Contributions from eastern Asia, India and Pakistan, represented by Hgs L-M76, Q-M378 and R2-M124, are detected in the Marsh Arabs, but at a very low frequency.

**Haplogroup J **accounts for 55.1% of the Iraqi sample reaching 84.6% in the Marsh Arabs, one of the highest frequencies reported so far. Unlike the Iraqi sample, which displays a roughly equal proportion of J1-M267 (56.4%) and J2-M172 (43.6%), almost all Marsh Arab J chromosomes (96%) belongs to the J1-M267 clade and, in particular, to sub-Hg J1-Page08. **Haplogroup E**, which characterizes 6.3% of Marsh Arabs and 13.6% of Iraqis, is represented by E-M123 in both groups, and E-M78 mainly in the Iraqis. **Haplogroup R1 **is present at a significantly lower frequency in the Marsh Arabs than in the Iraqi sample (2.8% *vs *19.4%; P < 0.001), and is present only as R1-L23. Conversely the Iraqis are distributed in all the three R1 sub-groups (R1-L23, R1-M17 and R1-M412) found in this survey at frequencies of 9.1%, 8.4% and 1.9%, respectively. **Other haplogroups **encountered at low frequencies among the Marsh Arabs are **Q **(2.8%), **G **(1.4%), **L **(0.7%) and **R2 **(1.4%).

### MtDNA variation

A total of 233 haplotypes [Additional file 2] and 77 sub-haplogroups (Table 1) have been identified in this survey. Only 26 of the observed sub-haplogroups are shared between the two populations, and most of the remaining are represented by singletons. According to their known or supposed geographic/ethnic origin [32-34], in addition to a strong West Eurasian component (77.8% and 84.1% in the Marsh Arabs and Iraqis, respectively), it is possible to recognize contributions from North/East and Sub-Saharan Africa and from East and South Asia.

**Table 1:** MtDNA haplogroup frequencies (%) observed in the Marsh Arabs in comparison to Iraqis.

**Haplogroups**^**a**^	Marsh Arabs (N = 145)	**Iraqis**^**b **^ (N = 176)	**Haplogroups**^**a**^	Marsh Arabs (N = 145)	** Iraqis**^**b **^ (N = 176)
**West Eurasia**	**77.8%**	**84.1%**	**West Eurasia (cont.)**		
***R0***	***24.1%***	***33.5%***	***N***	***15.1%***	***6.8%***
R0	0.7	-	*I*	*0.7%*	*1.7%*
R0a	6.9	4.0	I	0.7	0.6
			I1	-	1.1
*HV*	*4.1%*	*12.5%*			
HV*	3.4	9.1	*N1*	*8.2%*	*2.3%*
HV0a	0.7	-	N1b1	4.8	2.3
HV1	-	3.4	N1c	3.4	-
					
*H*	*12.4%*	*17.0%*	*W*	*4.8%*	*1.1*%
H*	7.6	10.8	W	2.7	1.1
H1	0.7	1.7	W4	0.7	-
H5	3.4	2.8	W6	1.4	-
H6b	-	0.6	*X*	*1.4%*	*1.7%*
H14a	0.7	1.1	X2	1.4	1.7
					
***KU***	***15.9%***	***19.4%***	**North/East Africa**	**2.8%**	**1.8%**
*K*	*6.2%*	*4.6%*			
K1	4.1	3.4	M1a	1.4	0.6
K1a8	2.1	0.6	M1a1	-	0.6
K2	-	0.6	M1b2	1.4	-
*U*	*9.7%*	*14.8%*	U6a	-	0.6
U	-	1.1			
U1a'c	-	2.3	**Sub-Saharan Africa**	**4.9%**	**9.1%**
U1b	-	0.6			
U2e	-	0.6	L0a1'4	0.7	-
U3	5.5	2.8	L0a2	-	1.1
U3a	-	0.6	L1b	1.4	-
U3b1a1	-	0.6	L1c2	-	0.6
U4	2.1	4.0	L1c3a	-	0.6
U5a1	1.4	1.1	L2a1'2	0.7	-
U5b3	0.7	-	L2a1	0.7	2.8
U9	-	1.1	L3*	0.7	-
			L3b	-	0.6
***JT***	***22.7%***	***24.5%***	L3e5	-	1.1
*J*	*15.2%*	*11.9%*	L3f	0.7	-
J1*	0.7	-	L3f1b	-	2.3
J1b	5.5	5.7			
J1b1b	-	0.6	**East Asia**	**1.4%**	**1.1%**
J1c	2.1	1.1			
J1d	0.7	1.1	B4	-	1.1
J1d1	-	1.1	B4c2	1.4	-
J1d2	-	0.6			
J2a	4.1	1.7	**Southwest Asia**	10.4%**	**4.0%**
J2b	2.1	-			
			M*	0.7	-
*T*	*7.6%*	*12.6%*	M33a2a	0.7	-
T1a	3.4	2.3	M37e	1.4	-
T1a1	-	0.6	R2	2.8	-
T1b	2.1	2.8	R5a	-	0.6
T2	-	2.3	U2d	-	0.6
T2a1b	-	1.1	U7	4.8	2.8
T2b	0.7	0.6			
T2c	-	2.3	**Others**	**2.8%**	-
T2c1	0.7	-	N*	0.7	-
T2e	0.7	0.6	R*	2.1	-

^a ^nomenclature according to van Oven and Kayser, built 12 (20 July 2011) [22].

^b ^included in the sample analysed by Al-Zahery et al. [12]; * indicates: groups of mtDNAs not belonging to any of the listed sub-haplogroups. For example, H* contains all H mtDNAs not belonging to the sub-clades investigated in this survey (H1, H5, H6, H14) ** value statically significant at p < 0.05.

**West Eurasian **mtDNAs observed in this study are approximately equally distributed into macro-Hgs **R0**, **KU**, and **JT**, although with haplogroup and sub-haplogroup differences between the two Iraqi samples. In the Marsh Arabs Hg **J **prevails (15.2%) followed by Hgs **H **(12.4%), **U **(9.7%) and **T **(7.6%). Conversely, in the control group, the most frequent is Hg H (17.0%) followed by Hgs U (14.8%), T (12.6%) and J (11.9%). Both the less represented **N1 **and **W **haplogroups show higher frequencies (marginally significant) in Marsh Arabs. The most frequent macro-Hg **R0 **includes molecules **R0a **((preHV)I), more represented among the Marsh Arabs (6.9% *vs *4.0%), **HV**, observed mainly as HV*, but especially **H **mtDNAs. Although the majority of the H mtDNAs (7.6% in Marsh Arabs *vs *10.8% in Iraqis) did not fall into any of the tested sub-haplogroups, a limited number of H subsets (H1, H5, H6, and H14) have been observed. In particular, while H5 (3.4% *vs *2.8%), H1 (0.7% *vs *1.7%) and H14 (0.7% *vs *1.1%) were found in both groups, H6 was observed only in one subject of the control group.

Almost all the main U sub-haplogroups and the nested K branch were found in the Iraqi sample, but only a sub-set of them (K1, U3, U4, U5, in addition to the South West Asian U7) were observed in the Marsh Arabs. The nested Hg K, mainly K1, was observed at a comparable frequency in both groups (6.2% in the marshes *vs *4.6%). The situation of macro-Hg JT is more complex. Significant differences (P < 0.05) emerged in the distribution of **J1 **and **J2 **sub-clades, with the latter much more frequent in the marshes (6.2% *vs *1.7%). By contrast, Hg T displayed a lower frequency in the marshes (7.6% *vs *12.6%) due to a significant lower incidence of its **T2 **sub-clade (2.1% *vs *6.9%, P < 0.05). On the other hand, Hgs **N1 **(8.2%) and **W **(4.8%), were both present in the marshes at a three-fold higher frequency than in Iraqis. Haplogroup X was detected as **X2 **with a frequency lower than 2% in both population samples.

**African haplogroups **are of North/East and sub-Saharan African origin and represent minor components in both groups. The North/East African contribution is mainly represented by Hg **M1 **which accounts for 2.8% of Marsh Arabs and 1.2% of the Iraqi sample, the latter displaying also 0.6% of Hg **U6**. The sub-Saharan African component comprised Hgs **L0**, **L1**, **L2 **and **L3 **and accounted for 4.9% in the marshes and 9.1% of the control sample. Out of the twelve African sub-haplogroups identified in this survey, six in the marshes and seven in the control sample, only one (L2a1) was shared between the two Iraqi groups.

The **Asian contribution **was significantly higher (P < 0.01) in the Marsh Arabs than in the control sample (11.8% *vs *5.2%). It includes mtDNAs belonging to the Southern Asian Hgs **M **(M*, M33, M37e) and **R2 **in Marsh Arabs, and **R5a **and **U2d **in the control sample. Haplogroup **U7**, frequent in Southwest Asia, was observed in both groups. The East Asian haplogroup **B4 **was detected at a very low frequency in both Iraqi groups.

### PC analyses

**Figure 3:**
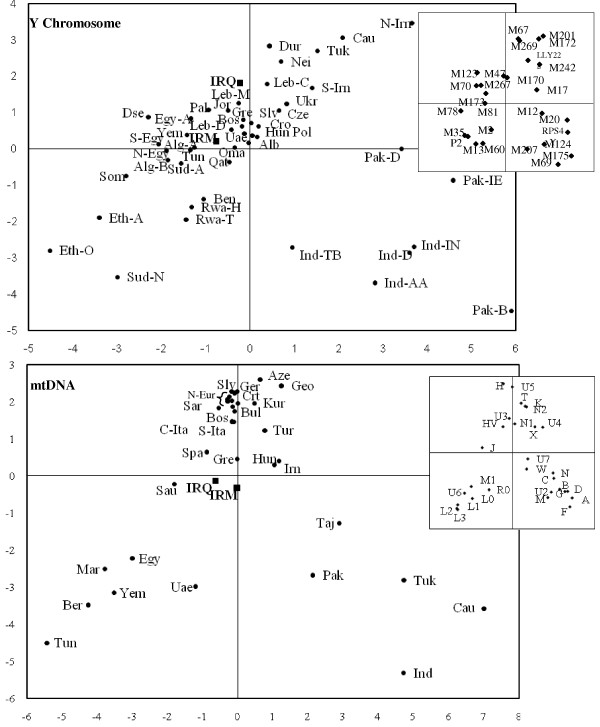
**Principal component analyses of Y-chromosome and mtDNA haplogroup frequencies**. The PCA analyses were carried out on haplogroups listed in Additional files 3 and 4. Haplogroups with frequencies lower than 5% in all the populations were not considered. On the whole, 28% of the total variance is represented for the Y-chromosome (16% by the first PC and 12% by the second PC) and 39% for the mtDNA (20% by the first PC and 19% by the second PC). Populations included are: IRM, Marsh Arabs; IRQ, Iraqi; Alb, Albania; Alg-A, Algeria-Arabs; Alg-B, Algeria-Berbers; Aze, Azerbaijan; Ben, Benin; Bos, Bosnia; Bul, Bulgaria; Cau, Caucasus; Crt, Crete; Cro, Croatia; Cze, Czech Republic; Dru, Druze; Egy, Egypt; Egy-A, Egypt-Arabs; S-Egy, South Egypt; N-Egy, North Egypt; Eth-A, Ethiopia-Amhara; Eto-O, Ethiopia-Oromo; Geo, Georgia; Gre, Greece; Hun, Hungary; Ind, India; Ind-AA, India-Austro-Asiatics; Ind-D, India-Dravidians; Ind-IN, India-Indo-Europeans; Ind-TB, India-Tibeto-Burmans; N-Eur, North Europe (Austria, Germany, Ireland, North Italy, Poland, Scotland); N-Irn, North Iran; S-Irn, South Iran; IRN, Iran; NeI, North East Italy; C-Ita, Central Italy; S-Ita, South Italy; Sar, Sardinia; Jor, Jordan; Kur, Kurds; Leb-C, Lebanon-Christians; Leb-D, Lebanon-Druze; Leb-M, Lebanon-Muslims; Mar, Morocco; Ber, Morocco-Berbers; Oma, Oman; Pak, Pakistan; Pak-D, Pakistan-Dravidians; Pak-B, Pakistan-Burushaski; Pak-IE, Pakistan-Indo-Europeans; Pal, Palestinian; Pol, Poland; Qat, Qatar; Rwa-H, Rwanda-Hutu; Rwa-T, Rwanda-Tutsi; Sau, Saudi Arabia; Slv, Slovenia; Som, Somalia; Spa, Spain; Sud-A, Sudan-Arabs; Sud-N, Sudan-Niloti; Taj, Tajikistan; Tun, Tunisia; Tur, Turkey; Tuk, Turkmenistan; Ukr, Ukraine; Uae, United Arab Emirates; Yem, Yemen (Details in Additional files 3 and 4).

In order to visualize the relationship linking Marsh Arabs with other Iraqis and surrounding neighbours [Additional files 3 and 4], principal component analyses of Y-chromosome and mtDNA haplogroups were carried out and the two PCs are illustrated in Figure 3.

For the Y-chromosome, the first two components, although accounting for only a quarter of the total variance, gather Marsh Arabs with almost all Arab populations and separate them along the first PC from western Eurasians, along the second PC from the African groups and by both components from South Asian populations.

When the PCA was based on mtDNA haplogroup frequencies, Marsh Arabs occupied, together with Iraqi and Saudi Arabian populations, a position in the middle of the plot among three distinct groupings: the first included western Eurasian, the second embraced all the South Asian groups while the third represented the North Africa and South Arabian Peninsula peoples.

For both systems, the longitudinal separation operated by the first PC is mainly due to the East-West decreasing frequency of East Asian haplogroups (see for example: Y-chromosome Hgs R2-M124, C-RPS4Y and H-M69; mtDNA Hgs A, F, D and G) and the increasing frequencies of the African haplogroups (see for example: Y-chromosome Hgs A-M13, B-M60, E-M35; mtDNA Hgs L1, L2 and L3) while the latitudinal separation operated by the second PC is mainly ascribable to the different distribution of haplogroups most frequent in West Eurasian (Y-chromosome Hgs J-M172, M267 and mtDNA Hgs H and U5), and the African-specific haplogroups (Y-chromosome Hgs A-M13, B-M60, E-M35 and mtDNA Hgs L0-3).

### Network analyses

**Figure 4:**
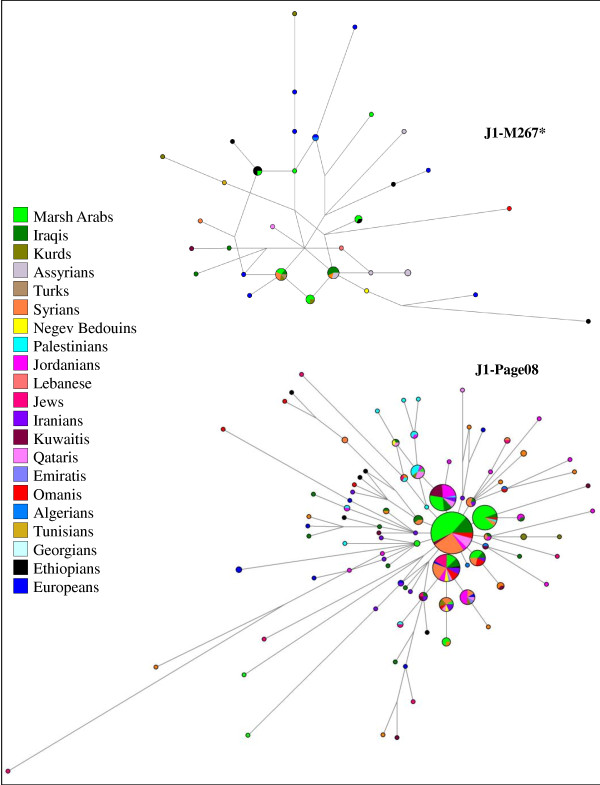
Networks of the STR haplotypes associated with haplogroups J1-M267* and J1-Page08, respectively. The eight STR (YCAIIa, YCAIIb, DYS19, DYS389I, DYS389II, DYS390, DYS391 and DYS392) haplotypes observed in 54 and 377 samples, respectively, are listed in Additional file 5. Circles and coloured sectors are proportional to the number of subjects, with the smallest circle and sector equal to 1. Connecting lines are proportional to the number of mutations.

Y-STR diversity at eight informative loci [Additional file 5] was used to evaluate the internal variation and phylogenetic relationships of J1-M267 Marsh Arab samples in comparison with neighbouring populations. Figure 4 illustrates the haplotype networks of paragroup J1-M267* and of the most frequent sub-lineage J1-Page08. While the J1-M267* network shows scarce structure, suggestive of a still heterogeneous clade, the J1-Page08 network displays a star-like shape centred around the most frequent haplotype of Marsh Arabs, which is shared with the majority of the Middle Eastern Arab groups. Signs of expansion were revealed by both networks but, in particular by that of J1-Page08 which, besides the central haplotype, includes at least three one-step derivatives overrepresented by Marsh Arab chromosomes. This expansion event of haplogroup J1-Page08 has a minor impact on the Iraqi control sample and the other Middle Eastern Arab populations.

**Figure 5:**
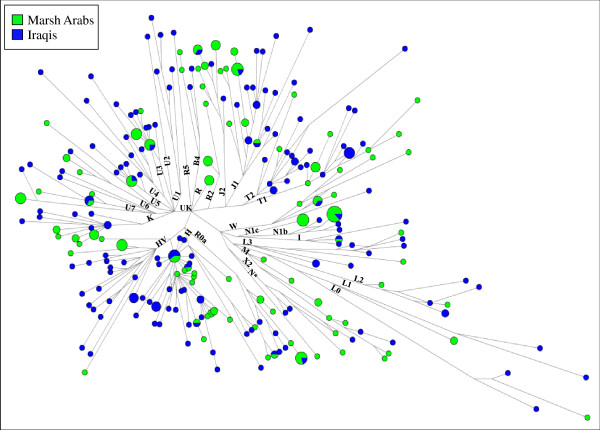
**Network of 233 mtDNA control-region haplotypes observed among 319 Iraqi samples**. These haplotypes [Additional file 2] refer to the variation observed between np 16024 and np 200. Circles are proportional to the number of subjects, with the smallest circle equal to 1. Connecting lines are proportional to the number of mutations including haplogroup diagnostic markers. Haplogroups and sub-haplogroups are labelled according to Table 1.

Figure 5 illustrates the network of the control-region mtDNA haplotypes associated with each haplogroup found in this survey [Additional file 2]. The majority of haplogroups were present in both population samples although with scarce sub-haplogroup and haplotype overlapping. In addition, differently from the control sample, a number of Marsh Arab haplotypes were shared between two or more subjects.

## Discussion

Two hypotheses have been proposed for the origin of Marsh Arabs: (i) they could be aboriginal inhabitants of Mesopotamia, correlated to the old Sumerians; (ii) they could be foreign people of unknown origin. Although the origin of Sumerians has yet to be clarified [5], the two main scenarios, autochthonous *vs *foreign ancestry, may have produced different genetic outcomes with Marsh Arabs being genetically closer to Middle Eastern groups or other populations, for instance those of the Indian sub-continent. Thus, in order to shed some light on this question Marsh Arab population was investigated for mtDNA and Y chromosome markers. Due to their characteristics (uniparental transmission and absence of recombination) and their wide datasets, they are, at present, among the best genetic systems for detecting signs of ancient migration events and to evaluate socio-cultural behaviours [35,36].

### Evidence of a Middle Eastern origin of the Marsh Iraqi Arabs comes mainly from the Y chromosome

Although different Western European mtDNA haplogroups were present in the Middle East in Palaeolithic times, they cannot always be interpreted as markers of Middle Eastern origin. For example, even if the mtDNA haplogroup H evolved in the Middle East ~18,000-15,000 years ago [34], different H sub-groups observed in this region, albeit at a rather low frequency, such as H1, arose outside and are most likely the result of gene flow from Europe [34,37].

Y-chromosome variation, like that of mtDNA, is highly geographically structured [34,38,39]. However, Middle Eastern haplogroup J, which accounts for the great majority of paternal lineages of this region and marks different migration events toward Europe, Africa and Asia, does not display, at present, evidence of back migrations.

### A common ancestral origin of Marsh Arabs and Southern Arabian peoples

Haplogroup J, with its two branches J1-M267 and J2-M172, is a Y-chromosome lineage dating to about 30,000 years ago. Its place of origin is still under discussion, but it is considered a landmark geographically linked to the Near Eastern region where the agricultural revolution and animal domestication appeared for the first time [34]. Accordingly, the frequency distribution of Hg J [13,40] shows radial decreasing clines toward the Levant area, Central Asia, the Caucasus, North Africa, and Europe from focal points of high frequency in the Near East [12,40,41]. Although both clades (J1-M267 and J2-M172) evolved *in situ *and participated in the Neolithic revolution, their different geographic distributions suggest two distinct histories. While J2-M172 has been linked to the development and expansion of agriculture in the wetter northern zone and is also considered the Y-chromosome marker for the spread of farming into South East Europe, J1-M267 has been associated with pastoralism in the semi-arid area of the Arabian Peninsula [42,43]. Despite this purported initial association, no evidence of pastoralism has been reported in the marsh area where one of the J1-M267 highest values (81.1%) has been observed (Additional file 6, Figure 6).

**Figure 6:**
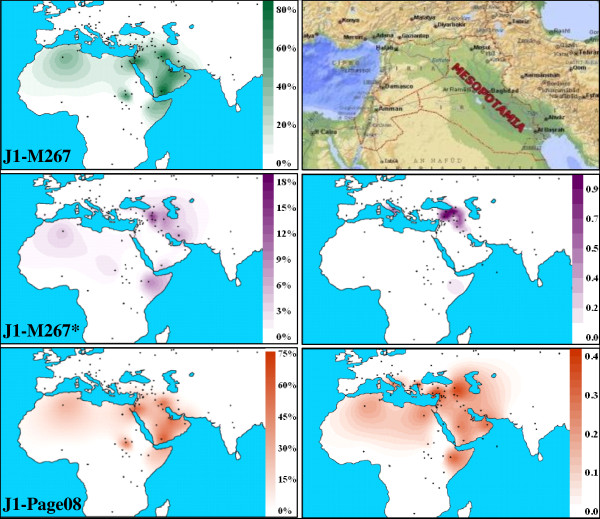
Frequency (left panels) and variance (right panels) distributions of Y-chromosome haplogroups J1-M267, J1-M267* and J1-Page08. Maps are based on 102 digit points [Additional file 6]. Variance data are relative to the microsatellite loci DYS19, DYS389I, DYS389II, DYS390, DYS391 and DYS392 typed in all the reported samples. Frequency and variance details are reported in Additional files 6, 7 and 8.

### Recent expansions shape the present Marsh Arab Y-chromosome landscape

When the two J1-M267 sub-clades, J1-M267* and J1-Page08 are considered (Figure 6), differential frequency trends emerge. The less represented J1-M267* primarily diffuses towards North East Mesopotamia and shows its highest incidence in the Assyrians of northern Iraq, and Turkey. By contrast, J1-Page08 accounts for the great majority of the J1 distribution in South Western Mesopotamia, reaching its highest value (74.1%) in the marsh area. By considering the STR haplotypes associated with the two branches, the highest values of variance are localized in northern Mesopotamia (North Iraq/South East Turkey) (Figure 6, Additional files 7, 8 and 9). For the J1-Page08 lineage, high variance values were also observed in Ethiopia, Oman and South Eastern Italy (Table 2). Although present data are not adequate to define the homeland of the J1-Page08 sub-clade, some useful information can be obtained from the haplotype network analysis (Figure 4). Thus, the pheripheric position of the Ethiopian and South Eastern Italian (European) haplotypes suggests that the high values of variance registered in these regions likely reflect the stratification of different migratory events, some of which occurred before the expansion and diffusion of the lineage outside the Middle Eastern area. As previously reported [31,41], also the value of variance in the Omani is affected by the concomitant presence of both pheripheric and centrally expanded haplotypes. In this context, the low variance (0.118) observed in the Marsh Arabs underlines a recent expansion involving few haplotypes, all of which occupying a central position in the J1-Page08 network (Figure 4). In the less frequent J1-M267* clade, only marginally affected by events of expansion, Marsh Arabs shared haplotypes with other Iraqi and Assyrian samples, supporting a common local background (Figure 4).

**Table 2:** Y-chromosome haplogroup J1-Page08 variance, divergence and expansion times based on six^(a) ^STR loci.

Population	N	Mean YSTR Variance	Divergence time ± SD kya	Expansion time (95% CI)		Reference
				Mean	Median	
Turkish/Area 4	5	0.367	12.1 ± 4.0			[43]
Turkish/Areas 6,5	7	0.294	9.5 ± 6.5			[43]
Turkish/Area 1	7	0.357	13.8 ± 3.7			[43]
Turkish/Area 9	5	0.150	6.0 ± 2.2			[43]
Assyrian	7	0.262	10.4 ± 5.2			[43]
Iraqi/Kurd	7	0.325	13.8 ± 6.5			[43]
Iraqi	41	0.154	5.9 ± 2.0	8.4 (1.9-20.1)	6.6 (1.7-17.9)	[Present study]
Iraqi/Marsh Arab	104	0.118	4.5 ± 2.6	4.8 (0.7-16.1)	3.5 (0.6-14.2)	[Present study]
Iraqi/Nassiriya	14	0.153	5.6 ± 2.9			[43]
Iranian/S-West	18	0.157	5.5 ± 2.0	8.1 (1.3-22.3)	5.9 (1.1-19.3)	[Present study]
Syrian	68	0.221	8.6 ± 2.9	9.8 (1.8-25.6)	7.3 (1.6-22.3)	[43]
Jordanian	35	0.234	9.3 ± 2.8	11.4 (2.2-30.2)	8.4 (2.0-26.4)	[43]
Palestinian	16	0.206	7.5 ± 3.7			[43]
Jewish	15	0.149	5.2 ± 2.6			[Present study]
Negev Bedouin	18	0.099	4.0 ± 1.9			[43]
Kuwaitian	16	0.221	11.0 ± 9.6			[Present study]
Qatarian	41	0.149	6.5 ± 2.2	6.6 (0.7-17.5)	5.1 (0.6-15.5)	[43]
Emirati	57	0.186	7.7 ± 2.1	8.9 (1.4-23.4)	6.6 (1.3-20.7)	[43]
Omanian	45	0.310	13.3 ± 4.4			[43]
Yemeni	42	0.205	8.8 ± 3.7			[43]
Egyptian	29	0.226	8.5 ± 2.6			[43]
Tunisian	19	0.158	5.9 ± 1.2			[Present study]
Algerian	6	0.189	6.0 ± 4.9			[Present study]
Ethiopian/Amhara	10	0.270	9.5 ± 2.7			[Present study]
Sudanese	26	0.091	3.5 ± 1.9			[31]
Italian	13	0.252	9.5 ± 3.6			[Present study]
Balkan/Central	7	0.171	8.1 ± 2.7			[14, Present study]

^(a) ^DYS19, DYS389I, DYS389II, DYS390, DYS391 and DYS392.

^(b) ^Divergence time according to [28,29].

^(c) ^Expansion time was estimated only in the populations involved in the J1-Page08 expansion as revealed by the network illustrated in figure 6, through the BATWING procedure [30], assuming an initial constant population size followed by expansion at time β with a growth rate of α. Alfa and beta parameters are as in [31].

For both methods the effective mutation rate and a generation time of 25 years [28] were used.

### Minor genetic influences in Marsh Arabs

Only a small proportion of the Marsh Arab gene pool derives from gene flow from neighbouring regions. On the paternal side, our phylogeographic data highlight some southwest Asian specific contributions as testified to by Hgs Q, L and R2, known as South Asian Y-chromosome lineages, primarily observed in India and Pakistan [29,44-47]. Different from the Iraqi control sample, the Marsh Arab gene pool displays a very scarce input from the northern Middle East (Hgs J2-M172 and derivatives, G-M201 and E-M123), virtually lacks western Eurasian (Hgs R1-M17, R1-M412 and R1-L23) and sub-Saharan African (Hg E-M2) contributions. On the other hand, the absence in both Iraqi groups of the North African E-M81 branch [13,48-50], speaks against substantial patrilineal gene flow from this region.

On the maternal side, a significant (East/Southwest) Asian component (11.8%) is present among Marsh Arabs as testified to by Hgs B4, M, R2 and U7. The B4 mtDNAs carry control-region motifs observed in Iran, Kirghizstan, Western Siberia, Vietnam, Korea [51-53] attesting to contact with Central and East Asia. This observation is likely due to recent gene flow, although it is worth noting that the ancient Silk Road passed through the Iraqi region from Basra to Baghdad. On the other hand, the majority of M, R2 and U7 mtDNAs display control-region motifs observed in South West Asian and in particular in India [47,54-57]. Additional evidence of the multiple relationships with South West Asia derives from the presence of one M33 mtDNA, which was completely sequenced, (GenBank accession number: JN540042). This mtDNA belongs to the M33a2a clade and clusters with three sequences, from Uttar Pradesh, Saudi Arabia [58] and Egypt [47], respectively. On the other hand, the presence in Iraq of Hgs M1 (in both Iraqi groups) and U6 (in the control sample) of North/East African origin [59] is indicative of some limited gene flow from that area. The sub-Saharan contribution is instead represented by haplogroups L0, L1, L2 and L3. It reaches values (~8%) in line with those reported for other Middle Eastern Arab populations [60,61].

### Gender differences in the Marsh Arab gene pool

In comparison with the control sample, representative of the general Iraqi population, Marsh Arabs are characterized by an important lower Y-chromosome heterogeneity (H_Y _= 0.461 *vs *0.887) whereas similar values of heterogeneity were observed for mtDNA (H_mtDNA_= 0.963 *vs *0.957). This is due to the presence of one prevalent Y-chromosome haplogroup, the J1-M267, which alone characterizes more than 80% of the Marsh Y-chromosome gene pool. Although patterns of lower male than female heterogeneity have been reported in many populations and usually ascribed to patrilocal residence [62-64], such a scenario can explain only part of the large difference observed in the geographically isolated marsh population. Among the different factors (e.g. polygamy, unequal male and female migration rates and selective processes) that can differently affect the extent of mtDNA and Y-chromosome heterogeneity, nonrandom-mating practices, common in the area, in association with cultural beliefs that support polygamy, may have contributed to cause the difference observed in the Marsh Arabs.

## Conclusions

The analyses carried out on the mtDNA and Y chromosome of the Iraqi Marsh Arabs, a population living in the Tigris-Euphrates marshlands, have shown: (i) a prevalent autochthonous Middle Eastern component both in male and female gene pools; (ii) weak South-West Asian and African heritages, more evident for mtDNA; (iii) a higher male than female homogeneity, mainly determined by the co-occurrence of socio-cultural and genetic factors; (iv) a genetic stratification not only ascribable to recent events. The last point is well illustrated by Y-chromosome data where the less represented J1-M267* lineage indicates Northern Mesopotamia contributions, whereas the most frequent J1-Page08 branch reveals a local recent expansion about 4,000 years ago (Table 2). Although the Y-chromosome age estimates deserve caution, particularly when samples are small and standard errors large, it is interesting to note that these estimates overlap the City State period which characterised Southern Mesopotamia, and is testified to by numerous ancient Sumerian cities (Lagash, Ur, Uruk, Eridu and Larsa).

In conclusion, our data show that the modern Marsh Arabs of Iraq harbour mtDNAs and Y chromosomes that are predominantly of Middle Eastern origin. Therefore, certain cultural features of the area such as water buffalo breeding and rice farming, which were most likely introduced from the Indian sub-continent, only marginally affected the gene pool of the autochthonous people of the region. Moreover, a Middle Eastern ancestral origin of the modern population of the marshes of southern Iraq implies that, if the Marsh Arabs are descendants of the ancient Sumerians, also Sumerians were not of Indian or Southern Asian ancestry.

## Authors' contributions

NAZ., OS and ASSB designed the research; NAZ and MAH performed the sample collection; NAZ, VB, VG generated the Y-chromosomal data; NAZ, MP, AO and BHK generated the mtDNA data; NAZ, VG and OS carried out the data analyses; NAZ, OS, AT, and ASS-B wrote the paper. All authors discussed the results, read and approved the final manuscript.

## Supplementary Material

Additional file 1 **Y-chromosome markers examined in this study**. The file provides information on the Y-chromosome markers examined in the present study. Click here for file

Additional file 2 **MtDNA control-region data of the samples reported in **Table 1. The file provides information about the mtDNA control-region haplotypes observed in the subjects of the present study. Click here for file

Additional file 3 **Absolute frequencies of Y-chromosome haplogroups and sub-haplogroups in the 48 populations included in the PCA**. The file provides the list of the populations and Y-chromosome haplogroups, along with their frequencies, used in the PCA analysis. Click here for file

Additional file 4 **Absolute frequencies of mtDNA haplogroups and sub-haplogroups in the 35 populations included in the PCA**. The file provides the list of the populations and mtDNA haplogroups, along with their frequencies, used in the PCA analysis. Click here for file

Additional file 5 Y-STR haplotypes associated with J1-M267* and J1-Page08. The file provides the Y-chromosome STR haplotypes used for the construction of the networks illustrated in Figure 4. Click here for file

Additional file 6 **Frequencies of Y-chromosome haplogroup J1-M267 and J1-Page08 from published sources and present study used for **Figure 6. The file provides the data used for constructing the maps illustrated in Figure 6. Click here for file

Additional file 7 **Y-chromosome haplogroup J1-Page-08 microsatellite variance from published sources and present study used for **Figure 6. The file provides the data used for constructing the map illustrated in Figure 6. Click here for file

Additional file 8 Y-chromosome haplogroup J1-M267* microsatellite variance from published sources and present study used for Figure 6. The file provides the data used for constructing the map illustrated in Figure 6. Click here for file

Additional file 9 **Y-chromosome J1 sub-haplogroups variance, divergence and expansion times based on six STR loci**. The file provides the variance, divergence and expansion times of the two J1 sub-haplogroups. Click here for file
